# Predictors and Long-Term Outcomes for Diffuse Large B-Cell Lymphoma (DLBCL) Patients Undergoing Surgery Prior to Systemic Therapy: A Nationwide Analysis

**DOI:** 10.7759/cureus.24448

**Published:** 2022-04-24

**Authors:** Urwat Vusqa, Thejus T Jayakrishnan, Veli Bakalov, Zena Chahine, Rodney Wegner, Cyrus Khan, Salman Fazal, Yazan Samhouri, Srikrishna V Malayala, John Lister

**Affiliations:** 1 Internal Medicine, Allegheny Health Network, Pittsburgh, USA; 2 Hematology and Oncology, Cleveland Clinic Taussig Cancer Center, Cleveland, USA; 3 Hematology and Medical Oncology, Allegheny Health Network, Pittsburgh, USA; 4 Hematology and Medical Oncology, University of Kentucky, Lexington, USA; 5 Radiation Oncology, Allegheny Health Network, Pittsburgh, USA; 6 Internal Medicine, Temple University Hospital, Philadelphia, USA

**Keywords:** systemic therapy, overall survival, time-to-initial therapy, surgery, diffuse large b-cell lymphoma

## Abstract

Background: A minority of patients diagnosed with diffuse large B-cell lymphoma (DLBCL) undergo surgery before the initiation of systemic therapy. The aim of this study is to explore the characteristics of patients undergoing surgery prior to systemic therapy (surgfirst), the predictors for surgfirst, and the survival outcomes.

Methods: The National Cancer Database was queried for patients with DLBCL diagnosed between 2006 and 2015, and we performed a subgroup analysis of patients that received surgfirst. Time-to-initial therapy (TTI) was defined as the time in days (d) from diagnosis to systemic therapy. Overall survival was measured from the day of diagnosis in terms of months (m).

Results: Factors associated with lower likelihood of surgfirst were non-Hispanic Black race (p-value<0.005), rural location (p-value<0.005), treatment at academic center (p-value<0.005), Medicaid insurance (p-value=0.01), comorbidity score >=3 (p-value 0.007), year of diagnosis, advanced stages of disease, and presence of B-symptoms. The TTI of systemic therapy was delayed in the surgfirst group - 34 (IQR 22-52) days vs. 23 (IQR 13-38) days, p-value<0.005. The five-year overall survival was 62.7% (95% CI 62.1-63.2%) vs. 58.3% (95% CI 57.7-60.0%) - HR 0.87 (95% CI 0.85-0.89), p-value<0.005. The factors associated with higher mortality were advanced comorbidities, lower educational status, disease primarily located in the bone, brain, and spinal cord, advanced clinical stage, presence of B-symptoms, and advanced age.

Conclusion: Despite the delay in systemic therapy, we could not identify a detrimental impact of surgfirst on survival. This needs to be confirmed in large-scale multicenter studies. We identified clinical and socioeconomic factors that affect treatment selection and survival.

## Introduction

Diffuse large B-cell lymphoma (DLBCL) is the most common type of non-Hodgkin's lymphoma (NHL), accounting for 22% of newly diagnosed NHL per year in the United States [1]. Chemoimmunotherapy with or without radiation therapy is recommended for the initial treatment of DLBCL [1]. A minority of patients undergo surgery before the initiation of systemic therapy for symptoms relief or treatment of complications of the disease. There are concerns about the delay in initiation of systemic therapy when surgery is performed, and the impact surgery has on long-term survival in this aggressive chemo-sensitive disease.

Data comparing outcomes for upfront surgery are largely limited to single-center studies on patients with DLBCL disease. In one study, the five-year relapse-free survival (RFS) was 86% in the chemotherapy group and 78% in the upfront surgery group (p=0.94). The five-year overall survival (OS) was 72.6% and 77.8%, respectively, which was not statistically significant (p=0.40) [2]. In another study, the five-year OS rates were 88% with surgery and 86% in patients treated with only chemotherapy (p=0.350) indicating that an operative approach was not advantageous [3]. These results were reproduced by others as well [4-6]. While not offering a significant survival advantage, significant morbidity associated with upfront surgery has been noticed especially when involving the GI tract. These include complications such as anastomotic leakage, gastrointestinal bleeding from remnant stomach or anastomosis site, dumping syndrome, and issues with malabsorption [4]. Given the similar outcomes and the morbidity associated with surgery, these studies recommend against surgery upfront. They recommend surgery to be reserved for the treatment of patients who present with disease complications, such as bleeding or perforation, or for palliation [7,8].

In the setting of limited data regarding upfront surgery for patients with DLBCL, data from National Cancer Database (NCDB) was used to produce a cohort study. We aim to explore the characteristics of patients undergoing surgery prior to systemic therapy (surgfirst), the clinical and socioeconomic predictors of upfront surgery, and their outcomes.

The abstract of this research was presented as a poster at the 62nd American Society of Hematology (ASH) Annual Meeting and Exposition (Virtual), December 5-8, 2020.

## Materials and methods

Sample data and model study

We conducted a retrospective cohort analysis using de-identified data accessed from the NCDB. The study was exempted from institutional review board (IRB) oversight and did not require ethics approval.

American Cancer Society and the Commission on Cancer of the American College of Surgeons established NCDB in 1989 through a joint program [9]. This comprehensive and expansive data set incorporates and parses records from greater than 1500 accredited hospitals. This helps to capture more than two-thirds of all incident cancers in the United States [10]. As per the memorandums signed and executed with accredited facilities, data from the Department of Defense, Veteran Affairs, and specific other programs are omitted from research files. The accreditation mandates a yearly 90% follow-up rate for all patients diagnosed within the last five years. To avoid censoring bias, survival outcomes are released after a minimum period of five years of follow-up.

To ensure the integrity of data and to avoid any duplicity, standardized algorithms are used. Variables include socioeconomic status, comorbidities, patient demographics, and the first course of therapy, defined as all treatment methods recorded in the treatment plan and administered to the patient before disease progression or recurrence. Treatments provided, discontinued or withheld due to progression, unsatisfactory response, or other therapy modifications caused by restaging or intercurrent events are not recorded. Furthermore, treatment durations or their dosage associated with specific chemotherapy regimens are not recorded.

**Figure 1:**
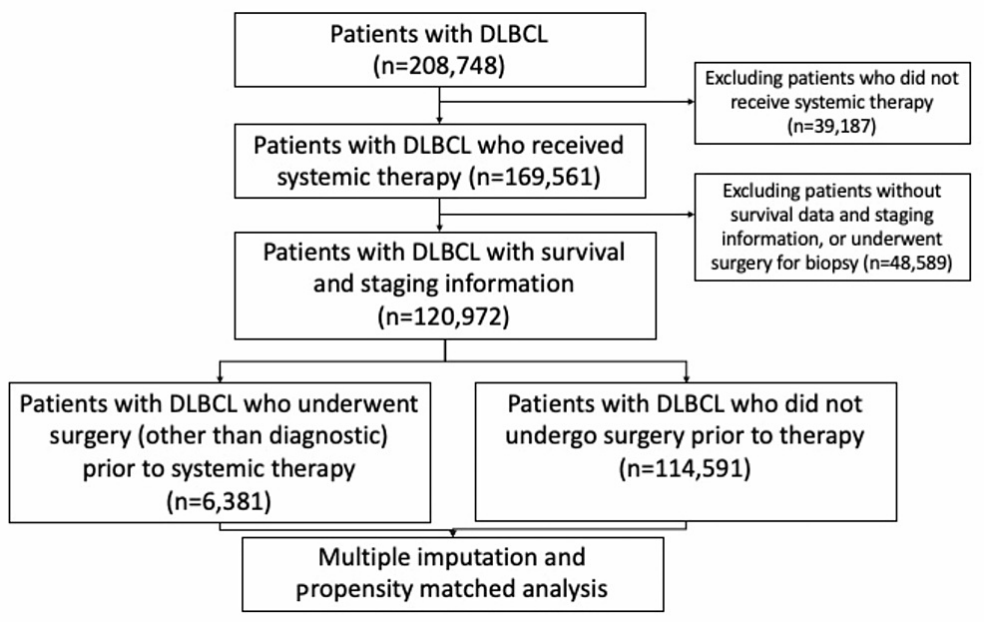
CONSORT diagram outlining the selection process for the study DLBCL: diffuse large B cell lymphoma; CONSORT: consolidated standards of reporting trials

The database was queried for patients with DLBCL (ICD-0-3 code 9680) diagnosed from 2006 to 2015. Inclusion criteria were patients that received systemic therapy as a first-line treatment and excluded patients with incomplete or missing data for stage, treatment characteristics, and survival. We excluded patients who underwent biopsy procedures for diagnosis and local procedures like tumor destruction or ablation (Figure 1).

Sociodemographic factors

Four categories were defined for 'race': Hispanics (H), non-Hispanic Whites, non-Hispanic Blacks, and others. Charlson/Deyo comorbidity index was used to capture comorbidity [11]. Sociodemographic factors were parsed from the residents’ census tract. The variable analyzed were gender (female and male) followed by literacy status represented in terms of percentage quartiles of pupils acquired no greater than high school education and median household income. The three location categories rural, urban and metropolitan were assigned as categorized by the United States Department of Agriculture Economic Research Service [12]. The next important factor was the insurance status. The NCDB database captures the insurance information from the patient admission factsheet and categorized insured or uninsured. NCDB makes use of the Commission on Cancer accreditation categorization of the facility. Disease staging and international prognostic staging system (IPSS) are incompletely reported in NCDB. Therefore, it was deemed unfit for analysis. The Commission on Cancer and the American College of Surgeons have neither verified nor been responsible for the methodology employed for the study performed, or inferences made from the data.

Statistical data analysis and their results

For this study, the primary outcome is OS, which was measured in terms of months (m) from the day of diagnosis. We also investigated time-to-initial therapy (TTI) which was defined as the time in days (d) from diagnosis to starting systemic therapy.

A comparison of baseline characteristics of varying treatment groups was utilized to form the basis of the descriptive statistics. Mean with standard deviation or median with interquartile range (IQR) methods were used to present continuous variables. Percentages and absolute numbers were then used to present categorical variables. In addition to that, t-test or ANOVA was used to compare the means of continuous variables. Percentages were compared using Fisher’s exact test or Pearson's chi-squared test. Analysis of the predictors for surgfirst was conducted using multivariate regression and represented as 95% confidence interval (CI) and odds ratio (OR). Propensity score-adjusted survival analysis was used to account for observed confounding factors. Propensity scores for surgfirst were developed by multivariable logistic regression to provide scores reflecting the conditional probability of surgfirst. The propensity model included only variables statistically significant on multivariable logistic regression and multiple imputations of variables were performed prior to propensity score matching. Survival estimates were performed using the Kaplan-Meier method. Subsequently, we constructed a Cox probability model adjusting for propensity score using inverse probability of treatment weights. The study was used to understand the impact on survival by significant independent variables, the same was used to report the hazards ratio (HR). Stata Statistical Software: Release 15 (2017; StataCorp, College Station, Texas, United States) was used for the analysis of the data. 95% confidence intervals and adjusted effect size estimates with 0.05 alpha level are reported to indicate statistical significance.

## Results

Out of 208,748 patients with DLBCL, 138,096 patients met the inclusion criteria. Of these, 6,381 (4.6%) received upfront surgery. The baseline characteristics of the study population are described in Table 1. The median age was 66 years (IQR 55-75) and 61.1% were males. Majority were non-Hispanic Whites, had a comorbidity score of 0, had private or Medicare insurance, and stage I disease. Most patients were treated in comprehensive community cancer centers. The top five extra-nodal disease sites were gastrointestinal (26.3%), male reproductive system (16.4%), endocrine system (4.1%), brain and spinal cord (6.4%), and head and neck (3.0%). The median follow-up was 47.6 months (IQR 14.0-78.9).

**Table 1:** Baseline characteristics of DLBCL patients DLBCL: diffuse large B-cell lymphoma; surgfirst: patients undergoing surgery prior to systemic therapy

Characteristic	Surgfirst (n=6381)	Not surgfirst (n=131,715)
Age in years	66 (55-75) years	66 (18-90) years
Last contact	47.6 (14.0-78.9) months	29.5 (9.2-62.7) months
Time to systemic therapy	34 (22-51) days	21.4 (11-36) days
Sex		
Male	61.1%	54.5%
Female	38.9%	45.5%
Race		
Non-Hispanic Whites	78.1%	77.1%
Non-Hispanic Blacks	5.6%	7.1%
Hispanic	6.4%	6.7%
Others	9.9%	9.0%
Insurance		
Uninsured	6.2%	5.9%
Private	40.0%	36.9%
Medicaid	4.6%	6.4%
Medicare	49.2%	49.8%
Median Income		
< $38,000	15.1%	15.6%
$38,000-$47,999	23.5%	23.1%
$48,000-$62,999	27.8%	27.5%
>=$63,000	33.7%	33.8%
Education		
≥21%	15.1%	15.8%
13.0-20.9%	24.6%	24.5%
7.0-12.9%	33.9%	33.6%
<7.0%	26.5%	26.0%
Comorbidity Score		
0	74.8%	73.9%
1	17.9%	17.6%
2	4.7%	5.0%
≥3	2.5%	3.5%
Distance to facility	Not different	
Location		
Metropolitan	83.6%	83.9%
Urban	14.7%	14.2%
Rural	1.7%	1.9%
Facility Type		
Community Cancer Center	9.1%	8.3%
Comprehensive Community Cancer Center	43.5%	40.2%
Academic/Research Program	33.9%	38.3%
Other	13.5%	13.2%
Year Group		
2006-2007	19.7%	15.0%
2008-2009	21.3%	17.7%
2010-2011	26.8%	20.0%
2012-2013	20.1%	22.3%
2014-2015	12.2%	25.0%
Stage		
Stage I	41.2%	23.2%
Stage II	23.5%	19.7%
Stage III	12.9%	20.1%
Stage IV	22.4%	36.9%
Presence of B-Symptoms		
None	77.4%	67.5%
Any B-symptoms	22.4%	32.1%
Pruritus	0.1%	0.2%
Both	0.2%	0.3%
Disease Sites		
Head and neck	3.0%	2.2%
GI	26.3%	7.9%
Respiratory system	2.5%	2.6%
Bone and joints	0.9%	2.4%
Soft tissue	0.6%	1.8%
Skin	0.6%	0.9%
Breast	0.3%	0.8%
Female reproductive system	1.85	0.3%
Male reproductive system	16.4%	0.4%
Urinary system	1.5%	0.5%
Eye and orbit	0.3%	0.2%
Brain and spinal cord	6.4%	5.0%
Endocrine including thymus	4.1%	1.2%
Lymph nodes	35.0%	71.0%
Mediastinum	0.3%	0.8%

Treatment selection

Results of multivariate regression analysis for upfront surgery in DLBCL patients are described in Table 2. Factors associated with a lower likelihood of surgfirst were non-Hispanic Black race (p-value<0.005), rural location (p-value<0.005), treatment at an academic center (p-value<0.005), Medicaid insurance (p-value=0.01), comorbidity score >=3 (p-value 0.007), year of diagnosis, presence of B-symptoms and advanced stages of disease. The TTI of systemic therapy was delayed in surgfirst group - 34 (IQR 22-52) days vs. 23 (IQR 13-38) days, p-value<0.005. The five-year overall survival was 62.7% (62.1-63.2) in surgfirst vs. 58.3% (57.7-60.0) in non-surgfirst - HR 0.87 (95% CI 0.85-0.89), p-value<0.005.

**Table 2:** Results for multivariate regression analysis for upfront surgery in DLBCL patients (used to generate propensity score-matched groups) DLBCL: diffuse large B-cell lymphoma

Characteristic	Odds Ratio (95% CI)	p-value
Age	0.992 (0.991-0.993)	<0.005
Sex		
Male	Reference	
Female	1.03 (1.01-1.05)	<0.005
Race		
Non-Hispanic Whites	Reference	
Non-Hispanic Blacks	0.93 (0.89-0.96)	<0.005
Hispanic	0.97 (0.94-1.01)	0.13
Others	0.99 (0.96-1.02)	0.61
Insurance Status		
Uninsured	Reference	
Private	1.16 (1.11-1.21)	<0.005
Medicaid	0.87 (0.82-0.91)	<0.005
Medicare	1.12 (1.08-1.17)	<0.005
Median Income		
< $38,000	Reference	
$38,000-$47,999	1.01 (0.99-1.05)	0.43
$48,000-$62,999	0.98 (0.95-1.02)	0.31
>=$63,000	0.96 (0.93-0.99)	0.02
Education		
≥21%	Reference	
13.0-20.9%	1.02 (0.99-1.05)	0.23
7.0-12.9%	1.04 (1.01-1.07)	0.011
<7.0%	1.05 (1.02-1.09)	0.004
Location		
Metropolitan	Reference	
Urban	1.00 (0.97-1.03)	0.999
Rural	0.88 (0.83-0.94)	<0.005
Comorbidity Score		
0	Reference	
1	0.96 (0.94-1.00)	0.75
2	0.97 (0.93-1.01)	0.09
>3	0.74 (0.70-0.78)	<0.005
Facility Type		
Community Cancer Center	Reference	
Comprehensive Community Cancer Center	0.96 (0.94-1.00)	0.02
Academic/Research Program	0.83 (0.80-8.86)	<0.005
Year Group		
2006-2007	Reference	
2008-2009	0.91 (0.89-0.94)	<0.005
2010-2011	1.06 (1.03-1.09)	<0.005
2012-2013	0.69 (0.67-0.71)	<0.005
2014-2015	0.34 (0.33-0.35)	<0.005
Stage		
Stage I	Reference	
Stage II	0.89 (0.87-0.91)	<0.005
Stage III	0.71 (0.69-0.73)	<0.005
Stage IV	0.56 (0.55-0.58)	<0.005
Presence of B-Symptoms		
None	Reference	
Any B-symptoms	0.79 (0.78-0.81)	<0.005
Pruritus	0.53 (0.42-0.67)	<0.005
Both	0.86 (0.71-1.04)	<0.005
Disease Sites		
Head and neck	4.16 (3.58-4.83)	<0.005
GI	11.87 (10.28-13.71)	<0.005
Respiratory system	3.17 (2.72-3.69)	<0.005
Bone and joints	1.20 (1.02-1.41)	<0.005
Soft tissue	1.18 (0.99-1.34)	0.06
Skin	2.18 (1.83-2.59)	<0.005
Breast	1.15 (0.95-1.40)	0.16
Female reproductive system	22.07 (18.83-25.85)	<0.005
Male reproductive system	145.53 (125.57-168.66)	<0.005
Urinary system	10.43 (8.91-12.22)	<0.005
Eye and orbit	3.89 (3.18-4.76)	<0.005
Brain and spinal cord	3.99 (3.44-4.62)	<0.005
Endocrine including thymus	10.08 (8.69-11.69)	<0.005
Lymph nodes	1.88 (1.63-2.17)	<0.005
Mediastinum	Omitted	

Factors identified in the regression analysis were utilized to perform propensity score-matched survival analysis as shown in Table 3. The factors associated with increased mortality were advanced comorbidities, lower educational status, disease primarily located in the bone, brain, and spinal cord, advanced clinical stage, presence of B-symptoms, and advanced age.

**Table 3:** Results for multivariate regression for survival in the propensity-matched pairs (no surgery 48,339 and surgery first 50,523)

Characteristic	Hazards Ratio (95% CI)	p-value
Age	1.040 (1.038-1.041)	<0.005
Sex		
Male	Reference	
Female	0.81 (0.79-0.84)	<0.005
Race		
Non-Hispanic Whites	Reference	
Non-Hispanic Blacks	0.98 (0.92-1.04)	0.550
Hispanic	0.81 (0.76-0.86)	<0.005
Others	0.95 (0.91 – 0.99)	0.018
Insurance Status		
Uninsured	Reference	
Private	0.75 (0.70-0.80)	<0.005
Medicaid	0.89 (0.81-0.98)	0.017
Medicare	0.996 (0.89-1.02)	0.21
Median Income		
< $38,000	Reference	
$38,000-$47,999	0.97 (0.93-1.01)	0.128
$48,000-$62,999	0.94 (0.90-0.98)	0.006
>=$63,000	0.91 (0.87-0.96)	<0.005
Education		
≥21%	Reference	
13.0-20.9%	0.98 (0.94-1.02)	0.342
7.0-12.9%	0.93 (0.89 – 0.98)	0.003
<7.0%	0.82 (0.78 – 0.87)	<0.005
Comorbidity Score		
0	Reference	
1	1.26 (1.22-1.29)	<0.005
2	1.53 (1.46-1.60)	<0.005
>3	2.05 (1.93-2.18)	<0.005
Facility Type		
Community Cancer Center	Reference	
Comprehensive Community Cancer Center	1.05 (1.00-1.10)	0.06
Academic/Research Program	0.95 (0.90-1.00)	0.06
Year Group		
2006-2007	Reference	
2008-2009	0.96 (0.92-1.00)	0.036
2010-2011	1.04 (1.00-1.08)	0.05
2012-2013	0.97 (0.93-1.01)	0.113
2014-2015	0.87 (0.82-0.91)	<0.005
Stage		
Stage I	Reference	
Stage II	1.15 (1.11-1.19)	<0.005
Stage III	1.30 (1.24-1.37)	<0.005
Stage IV	1.57 (1.52-1.62)	<0.005
Presence of B-Symptoms		
None	Reference	
Any B-symptoms	1.19 (1.15 – 1.23)	<0.005
Pruritus	0.39 (0.21-0.73)	0.003
Both	1.28 (0.98-1.58)	0.065
Upfront surgery	0.87 (0.85-0.89)	<0.005

**Figure 2:**
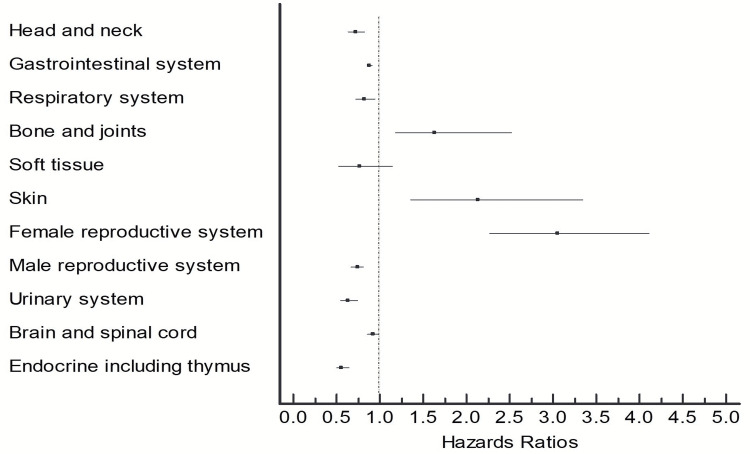
Survival outcomes for propensity score-matched groups

As described in table 4 and represented in Figure 2, the impact of surgery on survival outcomes varied according to the primary location of the tumor and surgical site as recorded in the database.

**Table 4:** Results for Cox regression for survival in the propensity-matched pairs demonstrating the impact of surgery by primary site

Disease Site	Univariate Hazards Ratio (95% CI)	p-value	Multivariate Hazards Ratio (95% CI)	p-value
Head and neck	0.67 (0.60-0.76)	<0.005	0.73 (0.64-0.83)	<0.005
GI	0.86 (0.84-0.90)	<0.005	0.87 (0.84-0.91)	<0.005
Respiratory system	0.68 (0.60-0.76)	<0.005	0.82 (0.72-0.94)	0.004
Bone and joints	1.94 (1.56-2.41)	<0.005	1.69 (1.23-2.32)	0.001
Soft tissue	1.40 (1.12-1.76)	0.003	0.73 (0.50-1.08)	0.119
Skin	0.94 (0.75 -1.18)	0.59	2.82 (1.68-4.77)	<0.005
Breast	1.18 (0.70-1.98)	0.53	n/a	
Female reproductive system	1.80 (1.46-2.22)	<0.005	3.06 (2.27-4.11)	<0.005
Male reproductive system	1.06 (0.97-1.15)	0.19	0.74 (0.67-0.81)	<0.005
Urinary system	0.65 (0.57-0.74)	<0.005	0.63 (0.54-0.74)	<0.005
Eye and orbit	1.04 (0.71-1.51)	0.85	n/a	
Brain and spinal cord	0.96 (0.91-1.02)	0.21	0.93 (0.86-0.99)	<0.005
Endocrine including thymus	0.53 (0.47-0.59)	<0.005	0.58 (0.51-0.66)	<0.005
Mediastinum	0.96 (0.45-2.03)	0.01	n/a	

## Discussion

Our study describes factors associated with increased mortality in patients with DLBCL. These include advanced comorbidities, lower educational status, primary disease located in the bone, brain, and spinal cord, advanced clinical stage, presence of B-symptoms, and advanced age. What our study, however, investigated in detail was whether a delay in systemic therapy due to upfront surgery impacted the OS for DLBCL patients; and what our study revealed was that despite the delay in systemic therapy due to surgery, there was no detrimental impact on survival which is comparable to OS in DLBCL of 64% [13].

We described the statistically significant factors that may be associated with upfront surgery. These factors include race, location, treatment center type, type of insurance, presence of co-morbidities, stage of disease, and presence of B symptoms. These were also explored in another study where stratified analysis showed that surgery achieved better survival in the male group, White and Black groups, married group, small intestine group, early-stage group, patients with B symptoms, as well as elderly patients (≥70 years old) (all p<0.05) [14]. However, the study conducted by Lin et al. failed to find an association between age, gender, race, and upfront surgery in DLBCL patients (due to the small sample size). Tumor location, cancer stage, prior chemotherapy, and radiotherapy were the only three factors that differed significantly between the two groups [15]. Another study did not find any significant differences between the two groups [16]. Hence it is safe to conclude that there is inconsistent data regarding if patients who undergo upfront surgery in DLBCL are truly different from those who do not.

An important addition to the present study was that we were able to demonstrate the heterogeneity of the outcomes by the site of the disease. We identified lower mortality among patients with the gastrointestinal system listed as the primary extranodal site of the disease. The improved outcome was also noted for the following systems or anatomic location - head and neck, respiratory system, male reproductive system, urinary system, brain and spinal cord, and endocrine system. On the other hand, survival outcome was worse for patients who underwent upfront surgery and had the following extra-nodal sites of disease - bone and joints, skin, female reproductive system. This finding is hypothesis-generating and needs to be investigated with large-scale studies where more disease-specific characteristics are available. There is limited data in the existing literature to support that the primary site of DLBCL plays a major role in indications for surgery and outcomes. While gastric lymphoma is usually treated with stomach preserving therapies, most patients with small intestinal lymphomas still undergo surgery before other therapies, as upfront surgery is associated with a lower risk of chemotherapy-induced perforation and bleeding [17]. Similarly, the role of surgery in primary central nervous system lymphoma (PCNSL) is still debated. A study involving PCNSL included 3342 patients collected from the Surveillance, Epidemiology, and End Results (SEER) database and demonstrated improved survival among patients who underwent surgery. However, further survival analyses demonstrated a benefit from surgery only among early‐stage patients [18].

Many studies that recommend against upfront surgery for DLBCL do so because of the morbidity and the mortality associated. Maor et al. reviewed 79 cases of primary gastric NHL (stages IE and IIE) and compared the survivors in each treatment group. They suggested that laparotomy and resection are associated with substantial mortality and morbidity, and accurate staging by laparotomy seldom changed treatment. Specifically, there were five perioperative deaths among 31 patients (16%) who underwent surgery, while there were no complications among the 35 patients treated with chemotherapy. The literature also showed a decrease in the role of primary surgery for primary gastric NHL [19].

Despite the evidence presented above, it is difficult to make a definitive recommendation as studies have also shown improved outcomes with upfront surgery. An early report from the University of Texas M.D. Anderson Cancer Center, Houston, Texas, United States, on this subject showed improved survival was observed in patients with stage I-IE and II-IIE aggressive lymphoma who underwent debulking surgery when the tumor was deemed resectable [18]. In a propensity-matched gastric DLBCL SEER study, The five-year cancer-specific survival rate of the surgical group was significantly higher than that of the conservative treatment group. Stratified subgroup analysis in the study showed that the survival difference was only significant among low-risk patients. It did not differ in the intermediate-risk and high-risk patients [15].

The limitations of the present study include unmeasured confounders from the retrospective study design. Using propensity score-matched methodology will decrease this bias. However, the propensity matching is limited by the data available in the database. Lack of details on disease characteristics for risk stratification, data on surgical complications, and status of disease recurrence are all limitations that will need to be addressed as well, as more data becomes available.

## Conclusions

Despite the delay in systemic therapy, we could not identify a detrimental impact on survival on all sites. It is possible that surgery truly does not significantly impact outcomes or that detrimental impact is limited to certain disease sites. Further evaluation of this practical question is warranted in large-scale prospective studies.
